# Comparison of treatment plans for hypofractionated high‐dose prostate cancer radiotherapy using the Varian Halcyon and the Elekta Synergy platforms

**DOI:** 10.1002/acm2.13380

**Published:** 2021-08-05

**Authors:** Jörg Tamihardja, Gary Razinskas, Florian Exner, Anne Richter, Patrick Kessler, Stefan Weick, Johannes Kraft, Frederick Mantel, Michael Flentje, Bülent Polat

**Affiliations:** ^1^ Department of Radiation Oncology University of Würzburg Würzburg Germany

**Keywords:** acute toxicity, dose evaluation, Halcyon, hypofractionation, prostate cancer, Synergy

## Abstract

**Purpose:**

To compare radiotherapy plans between an O‐ring and a conventional C‐arm linac for hypofractionated high‐dose prostate radiotherapy in terms of plan quality, dose distribution, and quality assurance in a multi‐vendor environment.

**Methods:**

Twenty prostate cancer treatment plans were irradiated on the O‐ring Varian Halcyon linac and were re‐optimized for the C‐arm Elekta Synergy Agility linac. Dose‐volume histogram metrics for target coverage and organ at risk dose, quality assurance, and monitor units were retrospectively compared. Patient‐specific quality assurance with ion chamber measurements, gamma index analysis, and portal dosimetry was performed using the Varian Portal Dosimetry system and the ArcCHECK^®^ phantom (Sun Nuclear Corporation). Prostate‐only radiotherapy was delivered with simultaneous integrated boost (SIB) volumetric modulated arc therapy (VMAT) in 20 fractions of 2.5/3.0 Gy each.

**Results:**

For both linacs, target coverage was excellent and plan quality comparable. Homogeneity in PTV_Boost_ was high for Synergy as well as Halcyon with a mean homogeneity index of 0.07 ± 0.01 and 0.05 ± 0.01, respectively. Mean dose for the organs at risk rectum and bladder differed not significantly between the linacs but were higher for the femoral heads and penile bulb for Halcyon. Quality assurance showed no significant differences in terms of ArcCHECK gamma pass rates. Median pass rate for 3%/2 mm was 99.3% (96.7 to 99.8%) for Synergy and 99.8% (95.6 to 100%) for Halcyon. Agreement between calculated and measured dose was high with a median deviation of −0.6% (−1.7 to 0.8%) for Synergy and 0.2% (−0.6 to 2.3%) for Halcyon. Monitor units were higher for the Halcyon by approximately 20% (*p* < 0.001).

**Conclusion:**

Hypofractionated high‐dose prostate cancer SIB VMAT on the Halcyon system is feasible with comparable plan quality in reference to a standard C‐arm Elekta Synergy linac.

## 1 INTRODUCTION

Hypofractionated^1^, ^2^ and ultra‐hypofractionated^3^, ^4^ external beam radiotherapy are novel treatment options for prostate cancer, one of the most common cancer types worldwide.^5^ Hypofractionation offers the benefit of reduced overall treatment time and possible improvement in the therapeutic ratio compared to conventional normo‐fractionated radiotherapy.^6^ For hypofractionated radiotherapy, highly conformal dose distribution and accurate dose delivery allow dose escalation with low risk of toxicity despite high biochemical relapse‐free survival.^7^ Advanced treatment options require high‐quality delivery and strict image‐guidance for verification. In this context Varian introduced the novel, closed system O‐ring Halcyon (Varian Medical Systems) linear accelerator which utilizes a 6 MV flattening filter‐free (FFF) beam with a double stack multi‐leaf collimator (MLC) which promises high delivery efficiency and consistent image‐guidance. The aim of this study was the comparison of prostate treatment plans calculated on two different treatment‐planning systems (TPS) in a multi‐vendor environment in terms of plan quality and the assessment of early acute physician‐reported toxicity. For this purpose, we analyzed Halcyon/Eclipse (Varian Medical Systems, Palo Alto, CA) treatment plans of hypofractionated prostate radiotherapy and compared them with the respective back‐up plans, optimized with Pinnacle^3^ TPS (Philips Radiation Oncology Systems) for irradiation on an Elekta Synergy Agility (Elekta AB) linear accelerator.

## 2 METHODS

### 2.1 Patient selection and dose prescription

In the course of commissioning the new Halcyon (Varian Medical Systems) linear accelerator at our institution, we calculated treatment plans for both Halcyon as well as Synergy as a back‐up solution before treatment start in case of technical difficulties. For this current study, the first twenty patients with prostate cancer, treated between April and July 2020 on the Halcyon, were retrospectively analyzed regarding the suitability of both machines for hypofractionated prostate radiotherapy. Synergy Agility plans served as back‐up plans but were not administered. All plans and back‐up plans were approved through institutional review. All patients had pathologically confirmed localized prostate cancer and received hypofractionated VMAT with daily kilovoltage cone‐beam CT.

**FIGURE 1:**
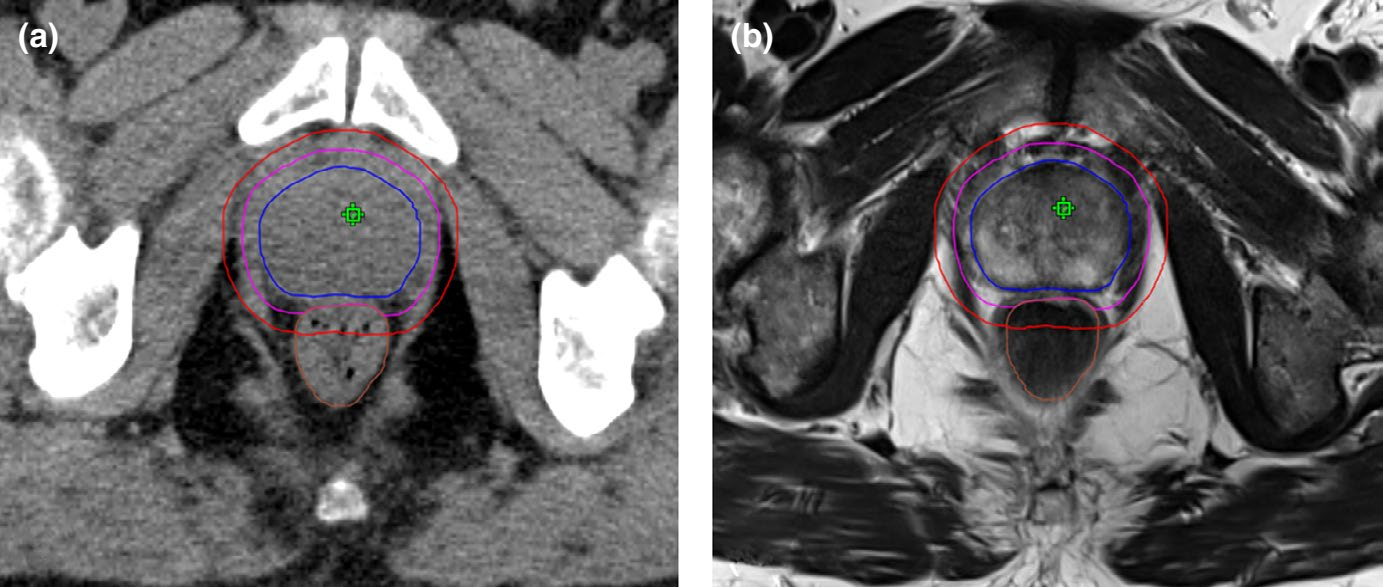
Target volume contours. Shown are the target volume contours of the CTV_P‐SV_ (blue), the high dose PTV_Boost_ (pink) and low dose PTV (red) in (a) the planning CT and (b) the planning MRI T2 sequence. The isocenter is marked in green. The low dose PTV was created by extending the CTV_P+SV_ (prostate with seminal vesicles) by 10 mm in all but dorsally directions, where a 7 mm margin was applied. The high dose PTV_Boost_ was created by extending CTV_P‐SV_ (prostate with only the base of the seminal vesicles) by 5 mm in all directions with avoidance of the organ at risk rectum

Patients were positioned in supine position with leg fixation and were instructed to have a full bladder and an empty rectum at each fraction. Radiotherapy was delivered in twenty fractions with a simultaneous integrated boost with two dose levels of 2.5 and 3.0 Gy per fraction. Overall treatment time was four weeks with five fractions per week. The prescribed PTV dose was 50 Gy (D_95%_) and 60 Gy to the PTV_Boost_ (D_Mean_). All patients had Magnetic Resonance Imaging for CTV delineation. The low dose PTV was created by extending the CTV_P+SV_ (prostate with seminal vesicles) by 10 mm in all but dorsally directions, where a 7 mm margin was applied. The high dose PTV_Boost_ was created by extending the CTV_P‐SV_ (prostate with only the base of the seminal vesicles) by 5 mm in all directions with avoidance of the organ at risk (OAR) rectum. According to institutional standard practice, fiducial markers were not utilized.^7^, ^8^, ^9^ The contouring concept is illustrated in Figure 1. Physician‐reported gastrointestinal (GI) and genitourinary (GU) toxicity was scored using Common Terminology Criteria for Adverse Events (CTCAE) v5.0 for all Halcyon patients. All patients signed an informed consent at hospital admission for retrospective data analysis.

### 2.2 Treatment planning

**FIGURE 2:**
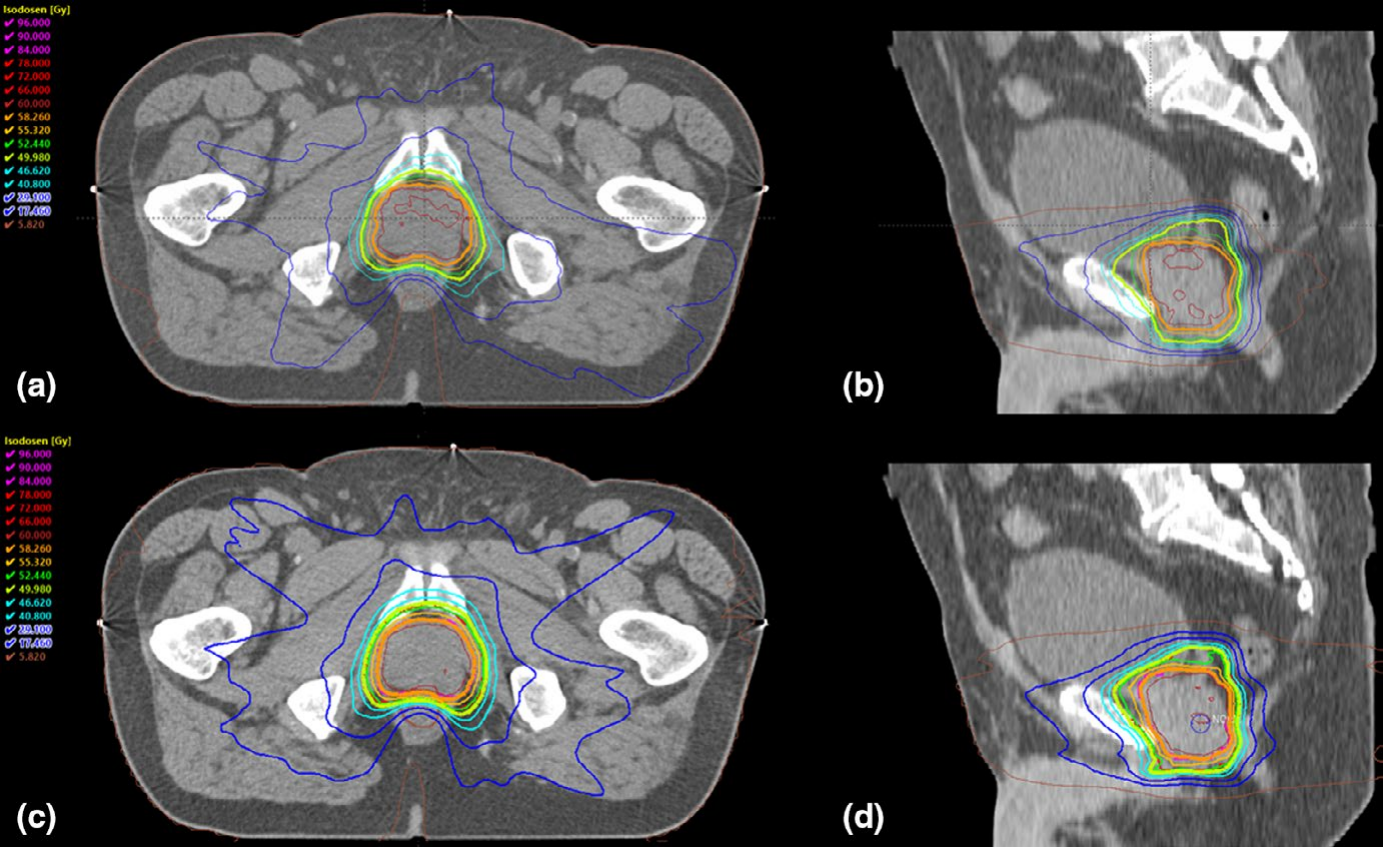
Dose distribution. (a) and (b) The dose distribution of the Halcyon prostate radiotherapy plan in the Eclipse TPS for patient 20. (c) and (d) The respective dose distribution of the same patient in the Pinnacle^3^ TPS for a Synergy Agility linac

For each patient, VMAT plans were generated for both delivery platforms with specifications defined according to clinical standards. Dose homogeneity was maintained by the standard deviation (SD) in PTV‐5 mm of less than 3%.^10^ Hypofractionated prostate radiotherapy treatment plans for the Synergy Agility linear accelerator were calculated with Pinnacle^3^ version 16.2 (Philips Radiation Oncology Systems), while for Halcyon plans Eclipse version 15.6 (Varian Medical Systems, Palo Alto, CA) was used. Plan optimization in Eclipse utilized the Photon Optimizer algorithm version 15.6.06 based on individual optimization objectives and weights (without knowledge‐based solution), whereas in Pinnacle its Auto‐Planning module version 16.2.1 was used to achieve the planning goals. Acuros External Beam version 15.6.06 (AcurosXB) with a grid size of 0.25 cm was used for dose distribution computation on the Eclipse TPS, while Pinnacle utilized the collapsed cone convolution algorithm and a grid size of 0.3 cm. An example of the resulting dose distribution is shown in Figure 2.

### 2.3 Halcyon linear accelerator

The Halcyon consists of a ring‐based linear accelerator with a 6 MV FFF photon beam, with a maximum dose rate of 800 monitor units (MUs) per minute and a maximum field size of 28 cm x 28 cm per isocenter. Two isocenters are combinable for an extended maximum treatment length of 36 cm. Halcyon 2.0 offers MV‐based cone‐beam computed tomography (MV‐CBCT) as well as kV‐based cone‐beam computed tomography (kV‐CBCT). The Halcyon has dual‐layer stacked–staggered MLCs with 10 mm leaf width. The gantry speed reaches up to four rotations per minute (RPM) and treatment is delivered at a maximum of 2 RPM. CBCT‐imaging before each fraction is mandatory for the Halcyon because there are no optical distance indicators or lasers available at treatment isocenter.

### 2.4 Synergy agility linear accelerator

The C‐Arm linear accelerator Synergy with an Agility head (Elekta AB) offers a maximum dose rate of 500 MUs per minute with a flattened beam profile, a maximum field size of 40 cm x 40 cm and interdigitating leaf pairs with a projected width of 5 mm at isocenter. In addition, kV‐CBCT and a variable photon beam energy of 6 MV, 10 MV, and 18 MV are available.

### 2.5 Plan evaluation

**FIGURE 3:**
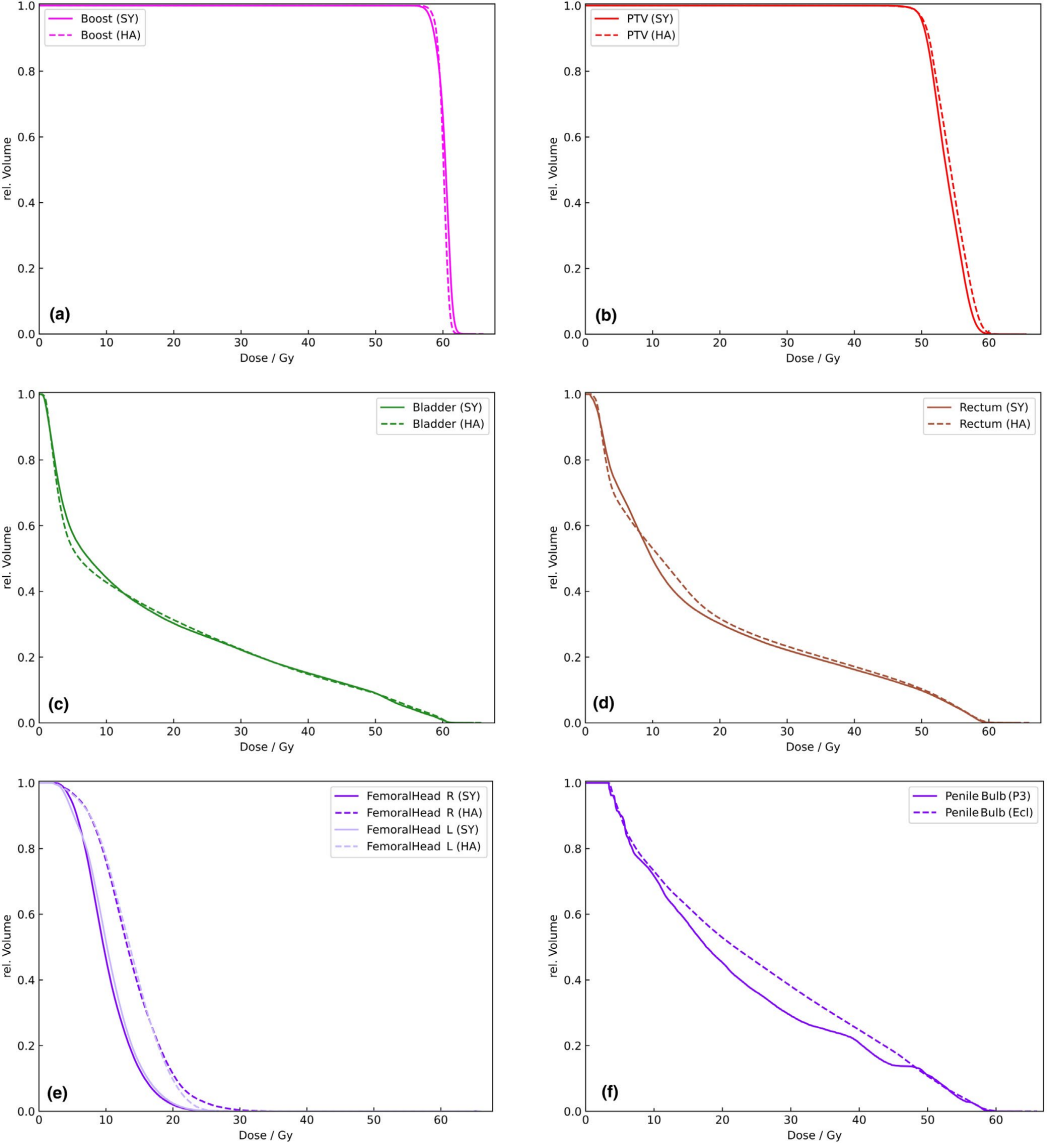
Average dose‐volume histograms. Shown are the average dose‐volume histograms of the target volumes (a) PTV_Boost_ and (b) PTV (without PTV_Boost_) and of the organs at risk (c) bladder, (d) rectum, (e) femoral heads, and (f) penile bulb for the studied cohort obtained for Synergy (solid lines) and Halcyon (dashed lines). SY, Synergy; HA, Halcyon

Retrospectively, the dose‐volume histograms (DVH) and dose parameters of each applied Halcyon plan (HA) were compared with the alternative Synergy Agility (SY) plan. Out of the individual dose volume histograms of the overall 40 patient plans average dose volume histograms for selected structures were calculated (Figure 3). Quantitative metrics for the organs at risk rectum, bladder, penile bulb, femoral heads as well as homogeneity and conformity indices for PTV_Boost_ and PTV (without PTV_Boost_) were evaluated.

The homogeneity index (HI) was defined as:

(1) $$\text{HI}=\left(D_{02\%}‐D_{98\%}\right)/D_{50\%}$$

The conformity index (CI) was defined as:

(2) $$\text{CI}=\left(\text{TV}*\text{PIV}\right)/{\text{TV}}_{\text{PIV}}^{2}$$

TV describes the target volume and TV_PIV_ the target volume within the prescription isodose volume and PIV the prescription isodose volume.^11^, ^12^ In addition, final plan MUs were analyzed for all plans.

### 2.6 Quality assurance (QA)

Patient‐specific quality assurance was performed for all Synergy and Halcyon plans with plan deliverance to an ArcCHECK^®^ phantom (Sun Nuclear Corporation) with the cavity plug inserted, equipped with a Semiflex 31010 ionization chamber (PTW‐Freiburg). Subsequent analysis of the dose measured on its diode cylinder was carried out using the software SNC patient version 6.7 (Sun Nuclear Corporation). For point dose measurements, passing criteria were percent dose deviation of less than 3% from the expected dose. Global gamma pass rates were evaluated for 3%/3 mm, 3%/2 mm, and 2%/2 mm criteria with a low‐dose threshold of 10% and a tolerance level of pixel passing of 98%, 96.5%, and 95%, respectively. Portal dosimetry was evaluated for each field for Halcyon plans with passing criteria of 2%/2 mm, 2%/1 mm, and 1%/1 mm and respective tolerable pass rates of 99.6%, 94%, and 87.5%.

### 2.7 Statistics

Matched‐pairs *t*‐tests were applied for the comparison of Synergy and Halcyon DVH and QA parameters for normally distributed parameters according to the Shapiro‐Wilk test if not indicated otherwise. In case of non‐normally distributed parameters Wilcoxon matched‐pairs signed rank tests were applied instead. Statistical significance was declared in case of a two‐sided *p*‐value <0.05. Statistical analysis was conducted using IBM SPSS v.26.0 (IBM Corp.). Quantitative values are expressed as mean ± standard deviation or as median with corresponding range as appropriate.

## 3 RESULTS

### 3.1 Patient characteristics

The median patient age was 69.2 years (59.3–84.2 years) and the median Karnofsky Performance Status was 90% (70%–100%). According to the risk classification of D`Amico 17 patients had intermediate‐risk and 3 patients high‐risk prostate cancer with a median initial prostate‐specific Antigen value of 7.00 ng/ml (1.03–24.90 ng/ml).^13^ Regarding histopathology, two patients had Gleason score (GS) 6, 10 patients GS 7a, 7 patients GS 7b, and 1 patient GS 8 prostate cancer. Fourteen out of 20 (70%) patients received androgen deprivation therapy.

Regarding acute toxicity, defined as toxicity occurring within the time period between start of radiotherapy and three months after the end or radiotherapy, no acute GI or GU toxicity higher than grade 2 was observed. For acute GI toxicity, three patients suffered from grade 2 proctitis (15%) and four patients showed mild grade 1 toxicity (20%). Thirteen patients (65%) showed no acute GI toxicity at all at first follow‐up 6 weeks after radiotherapy. For acute GU toxicity, 17 patients (85%) showed grade 1 GU toxicity with mild urinary urgency/non‐infective cystitis being the most reported toxicity. Three patients (15%) reported grade 2 GU toxicity with one case of grade 2 urinary obstruction and two cases of non‐infective cystitis. Regarding early late toxicity in the follow‐up after 6 months, GI toxicity was low with two cases of grade 1 toxicity out of 14 evaluable patients (14%). No early late GI toxicity higher than grade 1 has been observed. Early late GU toxicity remained moderate with 9 out of 14 cases of grade 1 toxicity (64%). One out of 14 patients (7%) suffered from grade 2 urinary obstruction. No higher than grade 2 toxicity has been reported at 6 months after the end of radiotherapy.

### 3.2 Target coverage

Mean D_Mean_ for PTV_Boost_ was not significantly different between both linacs with 60.2 ± 0.3 Gy and 60.0 ± 0.2 Gy for SY and HA (*p* = 0.052) and all plans were within planning tolerance range of ±1% for PTV_Boost_ D_Mean_. Also, all plans were within planning tolerance range of ±2% for PTV_Boost_ D_95%_.

Mean D_95%_ for PTV (without PTV_Boost_) was significantly different with 50.1 ± 0.4 Gy and 50.5 ± 0.3 Gy for SY and HA (*p* = 0.004) and all plans except one were within planning tolerance range of ±2% for PTV (without PTV_Boost_) D_95%_. One HA plan marginally missed the tolerance range with 2.1% above planning target. Furthermore, the planning objectives PTV_Boost_ D_99%_ >55.0 Gy; PTV_Boost_ D_01%_ <63.0 Gy and PTV (without PTV_Boost_) D_99%_ >46.7 Gy were reached in all cases. For both treatment systems, homogeneity was excellent with a median HI for PTV_Boost_ of 0.06 (0.05 to 0.09) and 0.05 (0.04–0.07) for SY and HA, respectively. Median CI for PTV_Boost_ was 1.17 (1.09–1.31) and 1.24 (1.09–1.54) for SY and HA, respectively. Target coverage parameters are summarized in Table 1.

**TABLE 1:** Target volume coverage and doses to OAR

	Synergy	Halcyon	*p*
PTV_Boost_			
D_98%_ (Gy)	57.8 ± 0.4	58.4 ± 0.4	0.001
D_02%_ (Gy)	61.7 ± 0.4	61.2 ± 0.3	<0.001
D_95%_ (Gy)	58.3 ± 0.4	58.8 ± 0.3	0.001
D_Mean_ (Gy)	60.2 ± 0.3	60.0 ± 0.2	NS
HI	0.07 ± 0.01	0.05 ± 0.01	<0.001
CI	1.17 ± 0.05	1.26 ± 0.10	<0.001
PTV (without PTV_Boost_)			
D_98%_ (Gy)	49.4 ± 0.5	49.4 ± 0.4	NS
D_02%_ (Gy)	58.3 ± 0.4	58.9 ± 0.7	0.002
D_95%_ (Gy)	50.1 ± 0.4	50.5 ± 0.3	0.004
D_Mean_ (Gy)	53.8 ± 0.3	54.4 ± 0.7	0.001
HI	0.16 ± 0.01	0.18 ± 0.01	0.018
CI	1.13 ± 0.02	1.10 ± 0.03	<0.001*
Rectum			
D_Mean_ (Gy)	17.4 ± 4.0	18.0 ± 4.5	NS
Bladder			
D_Mean_ (Gy)	16.2 ± 6.3	16.1 ± 6.1	NS
Femoral Head left			
D_Mean_ (Gy)	10.6 ± 2.9	13.6 ± 2.7	0.002
Femoral Head right			
D_Mean_ (Gy)	10.3 ± 2.6	13.7 ± 3.3	0.002
Penile Bulb			
D_Mean_ (Gy)	22.7 ± 12.2	25.2 ± 12.1	0.038

Target volume coverage and dose to organs at risk in Synergy Agility and Halcyon plans. Stated values indicate mean ± standard deviation. Statistical significance was defined as a two‐sided *p* < 0.05. Statistical significance was tested by matched‐pairs *t*‐tests in case of normally distributed parameters. In case of non‐normally distributed parameters, Wilcoxon matched‐pairs signed‐rank tests (*) were applied instead. OAR, Organ at risk; HI, Homogeneity Index; CI, Conformity Index; NS, Not significant.

Monitor units were significantly different between SY and HA plans. Median MUs were 854.1 (640.6–1039.9) and 964.1 (850.8–1553.0) for SY and HA, respectively (Wilcoxon matched‐pairs signed‐rank test, *p* < 0.001). An overview on MUs is given in Table 2.

**TABLE 2:** Treatment plan characteristics

Patient	Energy		Arcs		Monitor Units	
	Synergy	Halcyon	Synergy	Halcyon	Synergy	Halcyon
1	10 MV	6 MV FFF	2	2	780.5	850.8
2	6 MV	6 MV FFF	2	2	842.0	1000.6
3	6 MV	6 MV FFF	2	2	866.1	1553.0
4	10 MV	6 MV FFF	2	2	834.2	999.6
5	10 MV	6 MV FFF	2	2	899.5	1291.5
6	6 MV	6 MV FFF	1	4	640.6	886.5
7	6 MV	6 MV FFF	2	2	792.5	904.6
8	10 MV	6 MV FFF	2	2	923.9	976.6
9	6 MV	6 MV FFF	2	2	895.0	1163.0
10	6 MV	6 MV FFF	2	2	924.4	856.5
11	6 MV	6 MV FFF	1	2	753.0	1413.0
12	6 MV	6 MV FFF	2	2	926.3	903.2
13	6 MV	6 MV FFF	1	2	715.1	864.8
14	10 MV	6 MV FFF	2	2	782,3	931.1
15	10 MV	6 MV FFF	2	4	951.6	1048.8
16	10 MV	6 MV FFF	2	2	1039.9	965.5
17	6 MV	6 MV FFF	1	2	710.0	877.0
18	6 MV	6 MV FFF	2	2	814.8	938.9
19	6 MV	6 MV FFF	2	2	867.8	1036.0
20	10 MV	6 MV FFF	2	2	948.5	962.6
***Mean***			***1.8***	***2.2***	***845.4***	***1021.2***
***SD***			***0.4***	***0.6***	***98.1***	***192.2***

Treatment plan configurations of Synergy Agility and Halcyon plans. Monitor units were significantly different between both treatment platforms (two‐sided *p* < 0.001; Wilcoxon matched‐pairs signed‐rank test). The mean and standard deviation (SD) for all patients is stated at the bottom.

### 3.3 Organs at risk

Mean D_Mean_ was not significantly different for the organs at risk rectum and bladder. Mean D_Mean_ of the femoral heads was significantly lower for SY than for HA. For the right side, mean D_Mean_ was 10.3 ± 2.6 Gy and 13.7 ± 3.3 Gy for SY and HA, respectively (*p* = 0.002). For the left side, mean D_Mean_ was 10.6 ± 2.9 Gy and 13.6 ± 2.7 Gy for SY and HA, respectively (*p* = 0.002). All plans were within the planning target of D_05%_ < 31.5 Gy. Mean D_Mean_ for the penile bulb was significantly lower for SY with 22.7 ± 12.2 Gy compared to 25.2 ± 12.1 Gy for HA (*p* = 0.038). In one case D_Mean_ exceeded the planning target of <40.0 Gy. Organ at risk dose parameters are summarized in Table 1.

### 3.4 Quality assurance

For patient‐specific quality assurance, the dose measured with the ion chamber inserted into the ArcCHECK phantom showed a median deviation of −0.6% (−1.7 to 0.8%) for the Synergy and 0.2% (−0.6 to 2.3%) for the Halcyon. All ion chamber measurements were in the tolerance range of <3% deviation from the expected dose. The gamma pass rate analysis of the dose to the diode array based on 3%/3 mm, 3%/2 mm, and 2%/2 mm criteria showed no statistically significant differences between Synergy and Halcyon plans. Median pass rate for 3%/2 mm was 99.3% (96.7 to 99.8%) for Synergy and 99.8% (95.6 to 100%) for Halcyon. All ArcCHECK gamma pass rates and ion chamber measurements are listed in detail in Table 3.

**TABLE 3:** Patient‐specific quality assurance

Patient	ArcCHECK Synergy (%)				ArcCHECK Halcyon (%)			
	PD	3%/3 mm	3%/2 mm	2%/2 mm	PD	3%/3 mm	3%/2 mm	2%/2 mm
1	−0.2	99.8	99.1	97.8	−0.2	99.8	99.8	99.1
2	−1.1	100	99.4	97.3	−0.1	100	100	98.7
3	−1.1	99.5	97.3	94.3	2.3	99.3	98.2	95.4
4	−0.3	100	99.7	99.3	−0.6	99.3	99.2	97.0
5	−0.8	100	99.8	98.7	0.8	99.8	99.8	97.7
6	−0.5	99.8	99.1	98.4	0.7	100	100	100
7	−1.7	99.8	99.6	98.1	0.2	99.8	99.6	97.1
8	−0.1	99.8	99.6	98.2	−0.4	100	99.8	98.8
9	−1.5	99.6	98.7	97.4	1.1	100	99.6	98.3
10	−0.9	100	99.1	97.5	−0.6	99.5	98.4	95.4
11	−0.8	98.6	96.7	94.4	1.6	97.6	95.6	90.4
12	−0.3	99.8	99.4	98.0	−0.5	100	100	98.5
13	−0.9	100	99.8	99.4	0.8	100	100	98.6
14	−0.4	99.6	99.4	98.3	0.4	99.8	99.8	98.3
15	−0.5	99.6	99.4	98.3	0.4	100	100	99.2
16	0.8	99.5	98.8	96.9	0.1	100	99.8	99.1
17	−1.6	99.8	99.4	98.3	0.1	100	100	98.9
18	−0.7	99.7	98.6	97.8	0.1	99.6	99.2	98.2
19	0.3	100	99.2	97.9	−0.2	99.4	99.2	96.0
20	0.4	99.5	98.1	95.9	0.3	99.0	97.3	94.3
***Mean***	***−0.6***	***99.7***	***99.0***	***97.6***	***0.3***	***99.7***	***99.3***	***97.5***
***SD***	***0.7***	***0.3***	***0.8***	***1.4***	***0.7***	***0.6***	***1.1***	***2.2***

Patient‐specific quality assurance with gamma‐index analysis and ion chamber measurements. Detailed are the ion chamber point dose (PD) and the gamma pass rates for 3%/3 mm, 3%/2 mm, 2%/2 mm. Quality assurance was performed utilizing the ArcCHECK^®^ phantom with the cavity plug inserted (Sun Nuclear Corporation, Melbourne, FL, USA). The mean and standard deviation (SD) overall patients is stated at the bottom.

Halcyon portal dosimetry median pass rate for 2%/2 mm was 99.9% (99.6%–100%), for 2%/1 mm 96.8% (94.1%–98.4%), and for 1%/1 mm 92.2% (88.3%–94.5%). The respective tolerated pass rates of 99.6%, 94%, and 87.5% were exceeded for all portal dosimetry measurements.

For patient 11 ArcCHECK gamma index analysis showed a borderline pass rate for Synergy and Halcyon 2%/2 mm, Halcyon 3%/2 mm, and Halcyon 3%/3 mm. Patient 20 showed a borderline pass rate for Halcyon 2%/2 mm. Nevertheless, the respective plans passed overall quality assurance as Halcyon portal dosimetry pass rates were above the tolerance threshold for the 2%/2 mm, 2%/1 mm, and 1%/1 mm criterion for all patients.

## 4 DISCUSSION

In this study, the Halcyon O‐ring linac/Eclipse TPS was compared with a Synergy Agility C‐arm linac/Pinnacle TPS in terms of plan quality, dosimetric differences, and quality assurance. To our knowledge, this is the first study to evaluate the Halcyon`s performance for hypofractionated high‐dose prostate radiotherapy using a simultaneous integrated boost in twenty fractions in a multi‐vendor setting. The aim of this study was to validate the ability of the new delivery system to generate clinically acceptable treatment plans for the upcoming and important field of hypofractionation. Meta‐analyses have shown non‐inferiority in tumor‐control compared to normo‐fractionated prostate radiotherapy and our institution is in the process of transitioning to hypofractionated regimes in clinical practice to reduce overall treatment time and patient burden especially for older patients with co‐morbidities.^14^, ^15^ We report a “real‐world” cohort of clinically accepted and irradiated prostate radiotherapy plans. Strengths of this study are the uniform dose prescription, target volume contouring, and uniform cohort of prostate cancer patients.

The data presented here demonstrate the ability of the new Halcyon linac to produce robust and reliable treatment plans for hypofractionated prostate radiotherapy and confirm earlier investigations for different cancer types and radiotherapy regimes.^16^, ^17^, ^18^, ^19^, ^20^, ^21^ The patient‐specific QA showed no statistically significant differences in diode array‐based gamma pass rate measurements between Synergy and Halcyon. Agreement between the measured and calculated dose was high for both linear accelerators with a maximum deviation of 1.7% and 2.3% in ion chamber point dose for Synergy and Halcyon, respectively. Portal dosimetry gamma pass rates were within tolerance levels for the 2%/2 mm, 2%/1 mm, and 1%/1 mm criterion for all Halcyon plans. Mean ArcCHECK gamma pass rates were in the range of literature reported values with Pokhrel et al. reporting mean 98.6 ± 1.5% and 98.3 ± 2.0% for the 2%/2 mm criterion in prostate stereotactic body radiotherapy for Halcyon VMAT and TrueBeam VMAT, respectively.^22^

Both Synergy Agility, as well as Halcyon linear accelerators, showed comparable plan quality with excellent homogeneity in PTV_Boost_ with a mean HI of 0.07 ± 0.01 and 0.05 ± 0.01, respectively. Monitor units were significantly different between both platforms with approximately 20% higher monitor units for the Halcyon. Flores‐Martinez et al. attributed this effect to the 6 MV FFF beam in Halcyon as compared with the flattened beam delivered by a C‐arm linac.^23^ Albeit statistical differences in target coverage were observed for PTV_Boost_ and PTV (without PTV_Boost_), absolute differences were negligible and well within the treatment planning objectives.

Statistically significant differences in median D_Mean_ could be demonstrated for the femoral heads and the penile bulb, although the actual clinical impact may be limited as all plans were clinically acceptable and passed the institutional review board. In the future work, it will be studied if differences in OAR dose sparing are attributable to individual optimization objectives and weights and decrease with increasing planning experience for the Halcyon. The most important OARs rectum and bladder showed no clinically relevant differences and both Synergy and Halcyon plans were evaluated as equally acceptable in OAR protection as well as target volume coverage. In addition, the applied Halcyon plans resulted in a low rate of acute and early late toxicity with three cases of each acute grade 2 gastrointestinal and genitourinary and one case of grade 2 genitourinary toxicity. As the CHIPP^1^ and PROFIT^24^ trials hinted on an increase in acute gastrointestinal toxicity further analysis of the toxicity of hypofractionated prostate radiotherapy seems warranted. Catton et al. reported 16.7% acute gastrointestinal toxicity grade ≥2 for the hypofractionated radiotherapy arm and only 10.5% in the standard fractionated arm.^24^ In a recent publication on the long‐term outcome of our institutional standard practice for moderately hypofractionated prostate radiotherapy, we reported acute GU toxicity grade ≥2 of 30.1% and acute GI toxicity grade ≥2 of 13.0%.^7^ Despite different fractionation schemes, the current study does not show excessive acute toxicity compared to these historical data and the data from large randomized trials.^21^

There are limitations of this study: In general, the Halcyon linac only offers 28 cm field length for a single isocenter, which may limit its usage in some radiotherapy indications. Partly, this may be overcome by applying a multi isocenter technique and thereby increasing the maximum field length. As this study investigated prostate‐only radiotherapy, single isocenter planning was sufficient. Furthermore, while of less importance in prostate‐only radiotherapy, more variable treatment areas may suffer from the absence of rotational shift error compensation. An evaluation of setup‐errors would have exceeded the scope of this study but will be in the focus of investigations in future analyses. One of the advantages of the Halcyon is a shorter treatment time because of the fast‐rotating O‐ring linac compared to a conventional C‐arm linac. This was not evaluated in this report, as other studies demonstrated a reduction in beam‐on time compared to conventional linear accelerators.^25^, ^26^ We report a “real‐world” cohort of applied Halcyon plans and the respective Synergy plans, which were calculated as back‐up solution before treatment. Differences in the number of arcs, energies, and structure interpolation of the two TPS/linac combinations may have introduced some uncertainties in the dose comparison. We acknowledge that inter‐planner variability may have had an impact on our analysis as the planners, although very experienced in IMRT planning and with the Pinnacle TPS, did have less knowledge on treatment planning in the Eclipse TPS after the installation of the Halcyon. Despite this, all plans were carefully inspected and approved by the institutional review board of senior physicians. Lastly, the retrospective character and small sample size of this study warrants further prospective confirmatory data collection.

## 5 CONCLUSION

In a multi‐vendor environment, both Halcyon, as well as Synergy Agility linear accelerators, produced clinically equivalent treatment plans for hypofractionated high‐dose prostate SIB VMAT. Treating prostate cancer in 20 fractions of 2.5/3.0 Gy each on the Halcyon system is feasible with low rates of acute toxicities and with the equivalent quality compared to a standard C‐arm Elekta Synergy Agility linac.

## CONFLICT OF INTEREST

No conflicts of interest.

## AUTHOR CONTRIBUTIONS

JT, MF, and BP designed the study. JT, GR, FE, AR, PK, StW, JK, and FM participated in the data collection and analysis. All authors performed a critical review of the manuscript and finally approved the manuscript.

## FUNDING INFORMATION

This publication was supported by the Open Access Publication Fund of the University of Wuerzburg.

## Data Availability

The data that support the findings of this study are available on request from the corresponding author. The data are not publicly available due to privacy or ethical restrictions.
